# The relationship between intrinsic motivation and psychological wellbeing in combat sport athletes: the mediating role of mental toughness

**DOI:** 10.3389/fpsyg.2026.1793186

**Published:** 2026-04-14

**Authors:** Mehdi Duyan, Fatma Özoglu, Gülcan Tekin, İlker Günel, Talip Çelik, Servet Reyhan, Gamze Ok, Jindong Chang

**Affiliations:** 1 Faculty of Sports Sciences, Inönü University, Malatya, Türkiye; 2 Faculty of Sports Sciences, Aǧri Ibrahim Çeçen University, Aǧri, Türkiye; 3 Department of Physical Education and Sports, Osmaniye Korkut Ata University, Osmaniye, Türkiye; 4 Malatya Vocational School of Property Protection and Security, Inönü University, Malatya, Türkiye; 5 Institute of Health Sciences, Department of Physical Education and Sports, Inönü University, Malatya, Türkiye; 6 School of Physical Education, Institute of Motor Quotient, Southwest University, Chongqing, China

**Keywords:** athletes, combat sport, intrinsic motivation, mental toughness, psychological wellbeing

## Abstract

**Introduction:**

This study aimed to examine the mediating role of mental toughness (MT) in the relationship between intrinsic motivation (IM) and psychological wellbeing (PWB) in combat sport athletes. Combat sport athletes face unique psychological challenges that can significantly impact their wellbeing, yet the mechanisms through which intrinsic motivation influences psychological wellbeing remain underexplored.

**Methods:**

The sample of this correlational survey study consisted of 489 combat sport athletes from university teams in Turkiye. Data were collected using the IM subscale of the “Sport Motivation Scale (SMS)”, the “Mental Toughness Scale (MTS),” and the “Psychological wellbeing Scale (PWB).” Relationships between variables were examined via correlation analysis, and the proposed mediation model was tested using Structural Equation Modeling (SEM) and the bootstrap method (5,000 resamples).

**Results:**

The analyses revealed that IM had significant direct effects on both PWB (β = 0.385, *p* < 0.001) and MT (β = 0.486, *p* < 0.001). MT was also found to be a significant predictor of PWB (β = 0.324, *p* < 0.001). Most notably, a significant indirect effect was identified, indicating that IM influences PWB through the enhancement of MT [b = 0.108, SE = 0.019, 95% CI (0.070, 0.145)]. The standardized indirect effect was β = 0.158.

**Discussion:**

The research demonstrates that IM supports the PWB of combat sport athletes both directly and indirectly by fostering MT. These findings suggest that programs designed to enhance the PWB of these athletes should target not only their motivational sources but also their resilience-building skills. Future research should employ longitudinal designs to establish causal relationships and examine the effectiveness of psycho-educational interventions targeting IM and MT in enhancing PWB among combat sport athletes.

## 1 Introduction

Competitive sports environments expose athletes to stress factors that require MT (Sarkar and Fletcher, 2014; Gustafsson et al., 2017). Combat sports (taekwondo, karate, judo, kickboxing, muaythai, etc.) bring with them a number of psychological challenges such as performance anxiety, fear of injury, and intense competitive pressure (Mojtahedi et al., 2023; Anastasiou et al., 2024). These challenges have a direct impact on athletes' psychological states and can affect their ultimate performance outcomes (Andrade et al., 2019; Silva et al., 2019; Rossi et al., 2022; Lee et al., 2024). A comprehensive analysis of these psychological factors is considered important in terms of athletes' capacity to cope with these obstacles (MT), their sources of motivation, and maintaining and improving their PWB (Limani and Bulica, 2024). In this context, combat sports stand out as a unique field that constantly tests participants' psychological coping mechanisms due to their high physical contact, injury anxiety, and performance pressure (Vertonghen and Theeboom, 2010; Ciaccioni et al., 2025).

IM, within the Self-Determination Theory (SDT) framework, refers to participation in an activity driven by inherent enjoyment, interest, and satisfaction, and is fueled by the fulfillment of basic psychological needs (autonomy, competence, relatedness) (Ryan and Deci, 2000; Teixeira et al., 2012). In combat sports, the desire to master technical skills and personal development are powerful sources of intrinsic motivation (Cao and Lyu, 2024; Mossman et al., 2024). The increase in IM stemming from the fulfillment of basic psychological needs supports the athlete's psychological readiness for competition and improves physical performance (Alkasasbeh and Akroush, 2025). Research shows that IM is positively related not only to performance quality and persistence but also to increased life satisfaction and overall PWB (Diener et al., 2009; Bücker et al., 2018). The Broaden-and-Build Theory (Fredrickson, 2001) provides a complementary framework for understanding the relationships among IM, MT, and PWB. According to this theory, positive emotions-such as enjoyment, interest, and satisfaction derived from intrinsically motivated activities-broaden individuals' momentary thought-action repertoires, encouraging them to explore, learn, and engage with their environment. Over time, this broadened cognitive and behavioral flexibility enables individuals to build enduring personal resources, including psychological resilience, coping skills, and social connections (Fredrickson, 2001, 2004, 2013). In the context of combat sports, athletes who experience positive emotions through intrinsic motivation (e.g., enjoyment of skill mastery, satisfaction from personal improvement) are more likely to approach challenges with curiosity and openness rather than fear or avoidance. This broadening effect facilitates the development of mental toughness by encouraging athletes to persist through difficulties, view setbacks as learning opportunities, and maintain focus under pressure (Løvoll et al., 2017). The accumulated psychological resources, including mental toughness, subsequently contribute to enhanced psychological wellbeing by promoting positive functioning, life satisfaction, and personal growth (Cohn et al., 2009). Thus, the Broaden-and-Build Theory explains how IM initiates a positive spiral that builds MT, which in turn sustains and enhances PWB. Furthermore, research suggests that training in combat sports can reduce aggressive tendencies, potentially serving as a key mechanism for enhancing wellbeing. This effect is attributed to the development of self-discipline and self-regulation through consistent practice, which helps practitioners better manage their impulses and emotional responses (Oh et al., 2025).

PWB is a multidimensional construct involving an individual's positive evaluation of life, finding meaning, and realizing personal potential (Ryff, 2014; González-Hernández et al., 2017). For athletes, this concept encompasses satisfaction derived from sports and the capacity to maintain a balanced life (Davis et al., 2021). In this relationship between IM and PWB, MT is thought to play an important mediating role. MT is defined as the capacity to remain resilient in the face of difficulties, pressure, and failure, to maintain focus, and to show perseverance (Gucciardi et al., 2015). Most importantly, MT is a psychological structure that enables athletes to remain determined, focused, confident, and calm when faced with high pressure or adverse situations (Zhang and Yu, 2025).

Combat athletes constantly face physical and psychological stressors inherent in training and competition, making MT an indispensable psychological resource for them (Sarkar and Fletcher, 2014; Predoiu et al., 2024). MT is thought to play an important role in both maintaining athletes‘ performance and protecting their PWB. Especially in high-stress environments such as combat sports, the effect of resilience on PWB may become even more pronounced (Martín-Rodríguez et al., 2024; Healey et al., 2025). The literature indicates that IM can foster MT (Hsieh et al., 2024), and that MT can contribute to the maintenance of PWB (Croom, 2022; Yu et al., 2024). An intrinsically motivated athlete may be inclined to view obstacles as learning opportunities. This can facilitate the development of MT in athletes (Katsantonis et al., 2023). MT is considered an important indicator of athletic performance (Demir et al., 2025). It has been found to reduce negative thoughts (rumination) in athletes (Yılmaz et al., 2025), and supports the strengthening of athletes' psychological structures (Zhang and Yu, 2025).

A review of the current literature reveals that studies on combat sports have largely focused on physiological adaptations and performance outcomes (Andreato et al., 2022; Muñoz-Vásquez et al., 2023; Linhares et al., 2024, 2025). However, the mechanisms by which these sports influence PWB-particularly the causal and mediating relationships between fundamental constructs such as IM, MT, and PWB-remain insufficiently elucidated. Although the role of variables such as emotional intelligence and self-esteem has been highlighted (Ying and Yang, 2025), a comprehensive and empirical model of how IM develops an athlete's MT and how this process ultimately contributes to psychological wellbeing is lacking. Therefore, in the context of combat sports, the mediating role of MT in the effect of IM on PWB points to an important research gap that needs to be systematically investigated.

While combat sports offer numerous psychological benefits, it is important to acknowledge that they do not invariably promote psychological wellbeing. Certain conditions within the combat sport environment may actually undermine athletes' psychological health. These include excessive training loads leading to overtraining syndrome (Kellmann et al., 2018), negative coaching behaviors and authoritarian coaching styles that thwart athletes' basic psychological needs for autonomy, competence, and relatedness-thereby reducing intrinsic motivation and increasing psychological distress (Bartholomew et al., 2011), performance failure and repeated losses leading to burnout and decreased self-esteem (Gustafsson et al., 2017), aggressive or violent training environments that may promote fear and anxiety rather than personal development (Vertonghen and Theeboom, 2010), and the pressure to maintain weight through unhealthy practices such as rapid weight loss, which can lead to eating disorders and psychological disturbances (Roklicer et al., 2020). Furthermore, the high-risk nature of combat sports may increase injury anxiety and fear of failure, which can negatively impact psychological wellbeing (Mojtahedi et al., 2023). Therefore, understanding the protective factors-such as intrinsic motivation and mental toughness-that buffer against these potential negative outcomes is crucial for developing effective interventions to safeguard and enhance athletes' psychological health.

Within the framework of SDT, there is a reciprocal complementary relationship between the cultivation of intrinsic motivation and life satisfaction. In this context, it is suggested that increased life satisfaction may reduce the risk of burnout, which is a significant threat for athletes (Zhang and Ma, 2025). Building on this, this study aims to investigate the effects of IM on PWB in combat athletes and, in particular, the mechanisms of the mediating role played by MT. The findings of this study are expected to provide an empirical basis for coaches, sports psychologists, and administrators in combat sports to design more personalized and comprehensive support programs aimed at developing athletes‘ motivational resources and MT. Therefore, the primary objective of this study is to examine the mediating role of MT in the relationship between IM and PWB among university students practicing combat sports. It will be tested whether athletes' intrinsic passion for the sport contributes to their overall wellbeing through the MT capacity they develop during challenging training and competition processes. To this end, the following hypotheses have been developed within the scope of the study's objective:

H_1_: There is a positive and significant relationship between IM and PWB.
H_2_: There is a positive and significant relationship between IM and MT.
H_3_: There is a positive and significant relationship between MT and PWB.
H_4_: MT plays a significant mediating role in the relationship between IM and PWB.

## 2 Materials and methods

### 2.1 Research model

**Figure 1:**
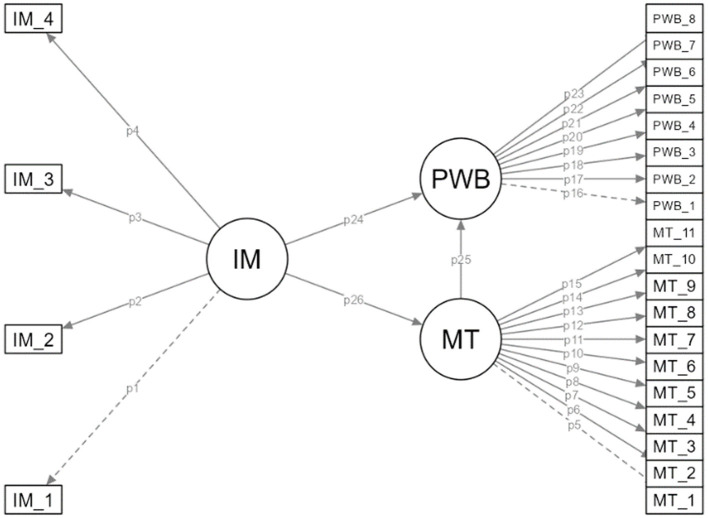
Research Model. IM, Intrinsic Motivation; MT, Mental Toughness; PWB, Psychological Wellbeing.

This study is a quantitative investigation based on a correlational survey model, aiming to examine the mediating role of MT in the relationship between IM and PWB in combat sport athletes. The correlational survey model is a research design intended to determine the level and direction of the relationship between two or more variables (Karasar, 2024). SEM was utilized to test the structural relationships among the variables. The hypothetical model of the research is illustrated in Figure 1.

### 2.2 Population and sample

The population of this study consists of licensed student-athletes actively competing in individual combat sports on university teams in Türkiye. The sample of the study comprises a total of 489 university combat athletes (253 male, 236 female) studying at various universities in Türkiye, selected using convenience sampling. The following inclusion criteria were established for participant selection: (a) being 18 years of age or older, (b) having trained regularly for at least two years, (c) training regularly at least 3 days a week, (d) being an active member of a university sports team. The exclusion criteria were as follows: a) having suffered a serious sports injury in the last six months, b) competing in a sport other than the specified combat sports (muaythai, kickboxing, karate, taekwondo, judo).

### 2.3 Data collection process and sample adequacy

Prior to commencing the data collection phase of the study, ethical approval was obtained from the Ethics Committee for Social and Human Sciences Research at Inönü University under protocol number 2025-25/2. The research was conducted in accordance with the ethical principles outlined in the Declaration of Helsinki. To collect data, online survey links were sent to combat sports athletes in university teams via their coaches. A total of 517 survey links were distributed, and responses were received from 496 (95.9% response rate). After the data were processed in an excel file, they were transferred to the Jamovi 2.7.6 statistical program. Subsequently, normality assumptions and outlier checks were performed on the data, and the final analyses were conducted using data from 489 participants. There are various opinions in the literature regarding sufficient sample size. For example, Tabachnick and Fidell (2001) consider a minimum of 200 participants necessary for SEM and indicate that larger samples are preferred. The sample in this study (N = 489) meets this recommendation. Additionally, a power analysis was conducted using the G^*^Power 3.1.9.7 program to determine statistical power. The analysis parameters were set at a 5% significance level (α = 0.05), a medium effect size (*f*^2^ = 0.15) as recommended by Cohen (2013), and 95% test power (1-β = 0.95) to minimize Type II error. Calculations based on the variables in the structural model indicated that the minimum sample size required for SEM analysis was approximately N = 119. Since the sample size of the current study (*N* = 489) exceeded this threshold and was consistent with the recommendations of other researchers, it was concluded that the sample size was sufficient for SEM statistical analysis.

### 2.4 Self-report measures

#### 2.4.1 Intrinsic motivation scale (IMS)

The Intrinsic Motivation (IM) subscale of the “Sport Motivation Scale-6” (SMS-6) Mallett et al. (2007), Turkish adaptation by (Demir, 2022), was used in this study. The SMS-6 originally consists of 24 items organized into six factors within the framework of Self-Determination Theory (Ryan and Deci, 2000): amotivation, identified regulation, external regulation, integrated regulation, introjected regulation, and intrinsic motivation. For the purpose of this study, which focused specifically on intrinsically determined reasons for sport participation (e.g., pure enjoyment, interest, and personal satisfaction), only the 4-item Intrinsic Motivation subscale was administered. Participants rated the items on a five-point Likert-type scale ranging from 1 (Strongly Disagree) to 5 (Strongly Agree). In the Turkish adaptation study, the IM subscale demonstrated good reliability with Cronbach's α = 0.77 and composite reliability CR = 0.78 (Demir, 2022).

#### 2.4.2 Mental toughness scale (MTS)

MT was assessed using an 11-item, single-dimensional scale developed by Madrigal et al. (2013) and adapted into Turkish by Erdogan (2016). Participants rated the items on a 5-point Likert scale ranging from 1 (Strongly Disagree) to 5 (Strongly Agree). Higher total scores reflect greater mental toughness. The Cronbach's alpha value for the Turkish adaptation of the scale was found to be 0.87 (Erdogan, 2016).

#### 2.4.3 Psychological wellbeing scale (PWBS)

The 8-item PWBS developed by Diener et al. (2009) and adapted to Turkish culture by Telef (2013) was used to assess PWB levels. Items are scored using a 7-point Likert scale ranging from “Strongly disagree” to “Strongly agree.” A high score on the scale indicates that the individual possesses many psychological resources and strengths. The internal consistency coefficient calculated by Telef (2013) for the scale is 0.80.

### 2.5 Data analysis

Data were analyzed using Jamovi 2.7.6. Normality was assessed through skewness and kurtosis values, which were within ±1.5, indicating normal distribution (Tabachnick et al., 2013). Descriptive statistics and Pearson correlation coefficients were computed. Confirmatory Factor Analysis (CFA) was conducted to evaluate construct validity, with model fit assessed using standard indices (χ^2^/df, RMSEA, CFI, TLI, SRMR; thresholds presented in Table 2). Reliability was examined using Cronbach's α and McDonald's ω, while convergent validity was assessed through Average Variance Extracted (AVE) and Composite Reliability (CR) (see Table 3). Structural Equation Modeling (SEM) with the bias-corrected bootstrap method (5,000 samples, 95% CI) was employed to test the hypothesized mediation model (Hayes, 2018). Statistical significance was set at *p* < 0.05.

## 3 Results

### 3.1 Descriptive statistics

The population of this study consisted of athletes participating in individual combat sports at university teams across Türkiye. The sample was formed by 489 university combat sport athletes selected through convenience sampling from various universities in Türkiye, all of whom participated voluntarily. Of the participants, 51.7% (*n* = 253) were male and 48.3% (*n* = 236) were female. Regarding age distribution, 50.1% (*n* = 245) were in the 22-25 age range, 31.3% (*n* = 153) were 26 years or older, and 18.6% (*n* = 91) were between 18 and 21 years old. In terms of sports discipline, Muay Thai accounted for the highest proportion at 41.7% (n=204), followed by Kickboxing (26.8%, *n* = 131), Taekwondo (14.9%, *n* = 73), Karate (11.7%, *n* = 57), and Judo (4.9%, *n* = 24). Regarding total sports experience, 87.9% (*n* = 430) of the participants had 7 or more years of experience, 8.2% *(n* = 40) had 6–7 years, 2.5% (*n* = 12) had 4–5 years, and 1.4% (*n* = 7) had 2–3 years. Additionally, 32.7% (n=160) of the participants were national team athletes. Detailed information regarding the demographic and sport-related characteristics of the sample is summarized in Table 1.

**Table 1:** Demographic characteristics of the athletes.

Variables		F	%
Gender	Male	253	51.7
	Female	236	48.3
	Total	489	100.0
Age	18-21 years	91	18
	22-25 years	245	50.1
	26 years and above	153	31.3
	Total	489	100.0
Sports Discipline	Judo	24	4.9
	Karate	57	11.7
	Taekwondo	73	14.9
	Muaythai	204	41.7
	Kickboxing	131	26.8
	Total	489	100.0
National Athlete	Yes	160	32.7
	No	329	67.3
	Total	489	100.0
Total Sports Experience	2-3 years	7	1.4
	4-5 years	12	2.5
	6-7 years	40	8.2
	7 years and above	430	87.9
	Total	489	100.0

### 3.2 Confirmatory factor and reliability analysis

The confirmatory factor analysis results indicated an acceptable fit between the measurement model and the data (see Table 2). The fit indices were all within acceptable ranges: χ^2^/df = 3.99, CFI = 0.987, TLI = 0.986, NFI = 0.983, RMSEA = 0.07, and SRMR = 0.06, indicating an acceptable model fit.

**Table 2:** Confirmatory factor analysis goodness-of-fit indices and reliability-validity values.

Fit index	Excellent fit criteria	Acceptable fit criteria	Obtained value	Result
χ^2^/df	0 ≤ χ^2^/df ≤ 2	2 < χ^2^/df ≤ 5	907/227=3.99	Acceptable
CFI	0.95 ≤ CFI ≤ 1.00	0.90 ≤ CFI < 0.95	0.987	Excellent
TLI (NNFI)	0.95 ≤ TLI ≤ 1.00	0.90 ≤ TLI < 0.95	0.986	Excellent
NFI	0.95 ≤ NFI ≤ 1.00	0.90 ≤ NFI < 0.95	0.983	Excellent
RMSEA	0.00 ≤ RMSEA ≤ 0.05	0.05 < RMSEA ≤ 0.08	0.07	Acceptable
SRMR	0.00 ≤ SRMR ≤ 0.05	0.05 < SRMR ≤ 0.08	0.06	Acceptable

CFA, Confirmatory Factor Analysis; CFI, Comparative Fit Index; TLI, Tucker-Lewis Index; NFI, Normed Fit Index; RMSEA, Root Mean Square Error of Approximation; SRMR, Standardized Root Mean Square Residual (Gürbüz, 2019; Ringle and Sarstedt, 2021).

### 3.3 Findings of reliability and convergent validity

According to the results presented in Table 3, the IM scale demonstrated acceptable reliability (α = 0.704, ω = 0.776) and convergent validity (AVE = 0.522 > 0.50). The MT scale exhibited excellent reliability (α = 0.928, ω = 0.935) and strong convergent validity (AVE = 0.680 > 0.50). For the PWB scale, reliability coefficients were found to be high (α = 0.873, ω = 0.880) and the convergent validity criterion was sufficiently met (AVE = 0.593 > 0.50). Consequently, it was concluded that the measurement model possesses values ranging from acceptable to excellent and is suitable for testing the structural model.

**Table 3:** Internal consistency and convergent validity indices of the scales.

Variable	Cronbach's α	Ordinal α	Composite reliability (ω1/ω_2_)	Hierarchical ω (ω3)	AVE
**IM**	0.704	0.752	0.776	0.846	0.522
**MT**	0.928	0.954	0.935	0.970	0.680
**PWB**	0.873	0.914	0.880	0.896	0.593

α, Cronbach's Alpha; ω, McDonald's Omega; AVE, Average Variance Extracted; CR, Composite Reliability. Acceptability thresholds: α & ω > 0.70, AVE > 0.50 (Gürbüz and Sahin, 2014; Gürbüz, 2019; Ringle and Sarstedt, 2021).

### 3.4 Correlation analysis

The results of the correlation analysis conducted to examine the relationships between the variables are presented in Table 4. The IM subscale consists of 4 items rated on a 5-point Likert scale, yielding a possible range of 4-20. The observed mean of 14.9 (SD = 3.38) corresponds to an average item score of 3.73 out of 5, suggesting that participants reported moderately high levels of intrinsic motivation. This finding is consistent with previous research using the SMS-6 in various athlete populations (Mallett et al., 2007), where mean scores for intrinsic motivation typically range between 14 and 16, indicating moderately high to high levels of intrinsic motivation among competitive athletes. According to the analysis results, positive and statistically significant relationships were found between all variables (p < 0.001). A moderate positive relationship was observed between IM and PWB (r = 0.421). The positive relationship between IM and MT was at a lower level (r = 0.308). Among the relationships examined, the strongest positive correlation was found between MT and PWB (r = 0.514).

**Table 4:** Pearson correlation coefficients among the study variables.

Variables	*M*	*SD*	1	2	3
1.IM	14.9	3.38	1		
2.MT	47.8	7.55	0.308^***^	1	
3.PWB	32.8	5.88	0.421^***^	0.514^***^	1

M, Mean; SD, Standard Deviation; IM, Intrinsic Motivation; MT, Mental Toughness; PWB, Psychological wellbeing.

^***^ *p* < 0.001.

Path analysis results are presented in Table 5. The findings indicate that an increase in IM has a statistically significant and positive effect on both MT and PWB.

**Table 5:** Standardized regression estimates and bootstrap confidence intervals for direct and indirect paths.

Direct effects								
Dep	Pred	Estimate	SE	LLCI (95%)	ULCI (95%)	β	***z***	***p***
PWB	IM	0.263	0.0338	0.196	0.329	0.385	7.77	< 0.001
PWB	MT	0.238	0.0339	0.172	0.304	0.324	7.02	< 0.001
MT	IM	0.452	0.0404	0.373	0.531	0.486	11.19	< 0.001
Indirect effect								
IM → MT → PWB		0.108	0.019	0.070	0.145	0.158	5.608	< 0.001

PWB, psychological wellbeing; IM, intrinsic motivation; MT, mental toughness; Dep, dependent variable; Pred, independent variable; Estimate, unstandardized regression coefficient; SE, standard error; LLCI, lower level confidence interval; ULCI, upper level confidence interval; β, standardized regression coefficient. The indirect effect and all 95% confidence intervals are based on 5,000 bootstrap samples.

**Figure 2:**
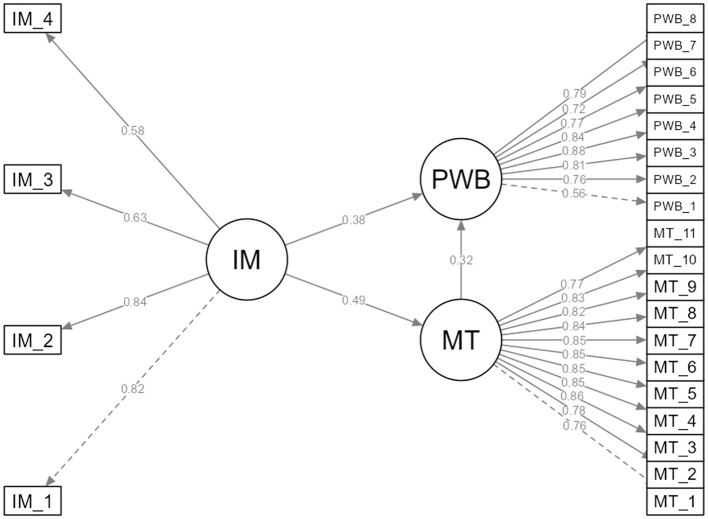
SEM Analysis Results. IM, Intrinsic Motivation; MT, Mental Toughness; PWB, Psychological Wellbeing.

The results of the SEM analysis are presented in Table 5. Findings regarding direct effects showed that IM was a positive and significant predictor of both PWB (β = 0.385, *p* < 0.001) and MT (β = 0.486, p < 0.001). Furthermore, MT was also found to be a significant predictor of PWB (β = 0.324, p < 0.001).The indirect effect was tested via bootstrap analysis (5,000 resamples). Results indicated that IM had a significant indirect effect on PWB through MT [b = 0.108, SE = 0.019, 95% CI (0.070, 0.145)]. Since the confidence interval did not include zero, the indirect effect was considered statistically significant. The standardized indirect effect was β = 0.158. These findings demonstrate that MT has a mediating role in the relationship between IM and PWB. The SEM illustrating these relationships is presented in Figure 2.

## 4 Discussion

This study aimed to examine the effect of IM on the PWB of combat sport athletes and the mediating role of MT in this relationship. The findings generally support the hypotheses constructed based on SDT. The analyses revealed that IM has both a direct effect on PWB and an indirect effect mediated through MT. The moderately high levels of intrinsic motivation observed in our sample align with previous research in combat sports. For instance, Cao and Lyu (2024) found that international practitioners of Chinese martial arts reported strong intrinsic motivation driven by personal development and skill mastery. Similarly, Mossman et al. (2024) demonstrated that autonomy-supportive environments in combat sports foster intrinsic motivation by satisfying basic psychological needs. Furthermore, studies on judo (Rossi et al., 2022), taekwondo (Lee et al., 2024), and mixed martial arts (Mojtahedi et al., 2023) have consistently reported that intrinsic motivation is a dominant motivational force among combat sport athletes, as the nature of these sports emphasizes personal growth, technical mastery, and self-referenced improvement rather than external rewards.

### 4.1 The effect of IM on PWB

The first primary hypothesis of the study was that IM would have a significant and positive direct effect on the PWB of combat sport athletes. Both correlation (*r* = 0.421) and structural model analysis (β = 0.385, *p* < 0.001) were found to support this hypothesis. According to SDT, the satisfaction of basic psychological needs-such as autonomy (the ability to make one's own choices as an athlete), competence (the feeling of mastering technical and tactical skills), and relatedness (forming meaningful social bonds with coaches and teammates)-is stated to be a fundamental source that enhances IM. In the specific context of combat sport athletes, the process of personal development during technical training (competence), having a say in competition strategies (autonomy), and the strong sense of camaraderie during training (relatedness) satisfy these basic needs, leading to an increase in their IM. This, in turn, promotes positive PWB by fostering elements such as positive affect, personal growth, and life satisfaction (Oh et al., 2025). For instance, Ying and Yang (2025) determined that participation in combat sports directly increased the PWB of university students.

The positive relationship between intrinsic motivation and psychological wellbeing observed in our study is well-grounded in self-determination theory, which posits that the satisfaction of basic psychological needs through intrinsically motivated activities directly enhances wellbeing (Ryan and Deci, 2000). This finding aligns with meta-analytic evidence demonstrating that intrinsic motivation is strongly associated with subjective wellbeing and life satisfaction across various populations (Diener et al., 2009; Bücker et al., 2018). In sport contexts specifically, autonomy-supportive environments that foster intrinsic motivation have been shown to promote athlete wellbeing by satisfying psychological needs for autonomy, competence, and relatedness (Mossman et al., 2024). Within combat sports, Rossi et al. (2022) found that higher motivation levels among judo athletes were associated with better psychological outcomes and performance. Similarly, recent studies on taekwondo (Lee et al., 2024) and mixed martial arts (Mojtahedi et al., 2023) have confirmed that intrinsically motivated athletes report higher levels of psychological wellbeing, as the enjoyment and personal satisfaction derived from training contribute to positive affect, personal growth, and life satisfaction.

Recent research has further elucidated the mechanisms linking motivation to wellbeing in combat sports. Zhang and Ma (2025) demonstrated that sport motivation negatively predicts athlete burnout through the chain mediating effects of life satisfaction and mental toughness, highlighting the protective role of intrinsic motivation against psychological exhaustion. Pekel et al. (2025) found that women engaged in martial arts demonstrated significantly higher psychological resilience in the sub-dimensions of control and challenge compared to non-practitioners, suggesting that combat sports participation enhances stress management skills and the ability to embrace difficulties. These findings align with our observation that intrinsically motivated athletes develop greater psychological resources Furthermore, Xu et al. (2026) demonstrated in a randomized controlled trial that university students engaged in combined skill- and spirit-oriented martial arts training showed significant improvements in flow states, positive psychological qualities, and courage compared to control groups. Their qualitative findings revealed that participants experienced enhanced intrinsic motivation, focus, sense of responsibility, and life perspective-suggesting that holistic martial arts training that integrates both technical and philosophical dimensions can foster the psychological resources that underpin wellbeing. Additionally, Levy and Boyd (2025) examined the psychology of weight cutting in combat sports, emphasizing the complex interplay between intrinsic motivation, body image expectations, and psychological risk factors, advocating for task-oriented strategies to promote healthier practices. Collectively, these findings suggest that intrinsically motivated athletes, who derive enjoyment and personal satisfaction from training, are better positioned to develop the psychological resources that contribute to positive affect, personal growth, and life satisfaction.

### 4.2 The effect of IM on MT

The second key finding of the research is that IM has a positive and significant predictive power on MT (β = 0.486, *p* < 0.001). Beyond their immediate ameliorative effect, the positive emotions triggered by IM can accumulate over time and permanently transform an athlete's approach to coping with stress and their way of evaluating negative situations (Løvoll et al., 2017). These positive emotional experiences stemming from IM in combat sport athletes may enable them to adopt more proactive coping strategies, develop their mental toughness capacity, and maintain motivational continuity in the face of encountered challenges (Ortiz-Franco et al., 2024). Empirical findings reveal that individuals who regularly experience intrinsically sourced positive emotions are more likely to exhibit higher resilience because these emotions activate cognitive reappraisal processes and adaptive behavioral responses to stress (Ahmed et al., 2025; Alkasasbeh and Akroush, 2025; Robazza et al., 2025). This evidence highlights that IM serves a fundamental function not only in creating enjoyable moments for combat sport athletes but also in the construction of MT and the support of sustainable PWB, thereby underscoring the critical importance of emotional engagement in this context (Oh et al., 2025).

The positive relationship between IM and MT observed in our study is consistent with a growing body of research in combat sports. For example, Hsieh et al. (2024) demonstrated in their systematic review and meta-analysis that intrinsic motivation serves as a key antecedent of mental toughness development across various sport disciplines, with effects persisting across different competitive levels and cultural contexts. In the context of combat sports specifically, Ortiz-Franco et al. (2024) found that intrinsically motivated martial artists exhibited significantly higher levels of resilience and were more likely to persist through challenging training sessions, viewing difficulties as opportunities for growth rather than threats to their competence. Similarly, Robazza et al. (2025) reported that athletes who derived enjoyment and satisfaction from their sport participation developed stronger coping mechanisms and emotional regulation skills, which are core components of mental toughness, through repeated exposure to challenging situations framed as learning opportunities.

Cross-cultural research has enriched our understanding of these processes. Dos Santos et al. (2025) examined the perspectives of 47 martial arts masters across Brazil, Portugal, and Spain, finding that instructors actively intervene when students lose self-control during combative situations, supporting the development of emotional regulation and resilience through structured pedagogical guidance. This aligns with phenomenological analyses demonstrating that combat sports training mediates dynamic transitions between affective, empathic, and motivational states, cultivating reflection and resilience through structured exposure to combative situations (Barreira et al., 2025).

Furthermore, de Lorenco-lima et al. (2025) found that more experienced Brazilian jiu-jitsu athletes exhibited significantly higher levels of resilience, self-control, and life satisfaction, suggesting that long-term engagement in combat sports cultivates the psychological resources necessary to manage frustration, pain, and physical challenges inherent in training. Jong et al. (2025) demonstrated in a large sample of runners that competitive athletes exhibit significantly higher levels of intrinsic motivation and mental toughness compared to recreational participants, suggesting that the IM-MT relationship transcends specific sport contexts and operates across different athletic populations.

These findings collectively suggest that the intrinsic enjoyment of skill mastery and personal growth in combat sports creates a sustainable psychological foundation upon which mental toughness is progressively built through cumulative positive experiences.

### 4.3 The effect of MT on PWB

The third significant finding of the study is that MT is a significant and positive predictor of PWB (β = 0.324, *p* < 0.001). Combat sports such as judo, karate, and taekwondo provide practitioners with both a structured means of stress management and an effective context for developing emotional regulation and impulse control. This multifaceted psychological utility underscores their role in promoting PWB (Oh et al., 2025). Tao and Li (2025) found that university students with combat sports experience exhibited higher levels of MT and self-control compared to their peers without such background. An experimental study determined that a 10-week combat sports-based intervention increased the psychological resilience of middle school students aged 12-14 (Moore et al., 2021). Similarly, Santana et al. (2022) reported that the mental toughness of combat sport athletes positively influenced their PWB. Therefore, MT is confirmed as a significant predictor of PWB. Correspondingly, elevated levels of MT have been associated with enhanced mental health and greater overall life satisfaction (Parsaiezadeh et al., 2024).

Recent experimental studies have provided further evidence for the psychological benefits of combat sports participation. Köroglu et al. (2025) demonstrated in a randomized controlled trial that 6 weeks of judo training significantly enhanced psychological resilience, self-control, and emotional expression in healthy males, supporting the notion that combat sports participation cultivates psychological resources essential for managing challenging situations. Similarly, Sahin et al. (2025) found that Muay Thai training significantly improved quality of life, self-control, and love of life, suggesting that engagement in combat sports fosters positive psychological outcomes that are closely aligned with psychological wellbeing. Pekel et al. (2025) further reported that women engaged in martial arts demonstrated significantly higher psychological resilience, particularly in managing stress and embracing challenges, compared to non-practitioners, highlighting the role of combat sports in developing psychological resources.

A comprehensive systematic review by Lin et al. (2017) established that mental toughness is consistently associated with positive psychological outcomes, effective coping strategies, and enhanced wellbeing across diverse populations. Longitudinal evidence from Maurin and Martinent (2023) further revealed that mental toughness positively predicts resilience, which in turn influences cognitive appraisals and perceived performance, highlighting the dynamic processes through which mental toughness contributes to psychological functioning. Additionally, a critical review by Lakicevic and Panfilova (2025) emphasized that psychological resilience is as important as physical conditioning in combat sports, with mental toughness serving as a key factor in alleviating performance anxiety and mitigating psychological stressors.

Collectively, these findings reinforce the conclusion that mental toughness, cultivated through combat sports participation, contributes to enhanced psychological wellbeing by fostering resilience, self-control, and positive life orientation.

### 4.4 The mediating role of MT and the holistic evaluation of the model

A primary and significant contribution of this study is the empirical demonstration, via Bootstrap analysis, that MT plays a significant mediating role in the relationship between IM and PWB in combat sport athletes. The analysis revealed that IM has a significant indirect effect on PWB through mental toughness [β = 0.158, 95% CI (0.070, 0.145)]. The fact that the confidence interval for this indirect effect does not include zero confirms its statistical reliability. This finding indicates that intrinsically motivated athletes' enjoyment of and interest in sports strengthens their capacity to cope with difficulties (MT), and this enhanced capacity, in turn, positively develops their overall PWB. This finding is consistent with other research in the literature. For example, Santana et al. (2022) determined that MT plays a similar mediating role in the relationship between subjective vitality and wellbeing. However, the present study specifically extends this mechanism to the context of combat sports with a focus on IM.

Our findings point to a process supported by SDT (Ryan and Deci, 2000) and the Broaden-and-Build Theory (Fredrickson, 2001). According to SDT, activities that satisfy the needs for autonomy and competence, such as combat sports, create a strong source of IM. Fredrickson's (2001) theory, on the other hand, posits that the positive emotions arising from this IM broaden an individual's thought-action repertoire, paving the way for the accumulation of long-term personal resources such as MT. Similarly, (Duyan and Selçuk 2024) stated that positive emotional experiences nourish the pool of psychological resources. It is also noted that MT, strengthened through this positive process, forms the foundation of an individual's stress coping capacity and plays a crucial function in preserving overall psychological functioning (Zhang and Yu, 2025). Consequently, the findings of this study support the validity of these theoretical frameworks in the combat sports context and provide empirical evidence for a relational model wherein IM leads to wellbeing through the pathway of MT.

When contextualized within the broader literature, our findings both align with and extend previous research. The positive relationship between IM and MT observed in this study (β = 0.486) is consistent with findings from individual sport contexts (Gucciardi et al., 2015) but appears stronger than those reported in team sport settings (Coulter et al., 2016), suggesting that the high-physical-contact nature of combat sports may amplify the motivational processes underlying mental toughness development. Recent systematic reviews have further confirmed the robust association between psychological resources and combat sports participation across diverse populations (Gross et al., 2018; Ciaccioni et al., 2025). Recent research in combat sports has specifically highlighted the importance of psychological resilience and imagery skills in managing emotions such as anger, demonstrating the critical role of mental skills in optimizing athletic performance and wellbeing (Di Corrado et al., 2025). Cross-culturally, while studies in Western individualist contexts have reported similar patterns regarding the positive associations between mental toughness and adaptive outcomes such as positive affect and performance (Mahoney et al., 2014), the magnitude of the mediation effect in this Turkish sample [b = 0.108, 95% CI (0.070, 0.145); standardized β = 0.158] suggests potential cultural nuances in how motivational resources translate into psychological wellbeing. For instance, collectivist cultural values may influence how athletes perceive and internalize the psychological benefits of intrinsic motivation (Markus and Kitayama, 1991). This finding aligns with recent cross-cultural research examining psychological resilience in combat sports practitioners across different sociocultural environments (Dos Santos et al., 2025; Predoiu et al., 2025; Spantios et al., 2025). Furthermore, comprehensive models of sport motivation emphasize the multidimensional nature of motivational processes, integrating intrinsic motivation with cognitive and environmental factors to explain athletic behavior (Tušak et al., 2022). Our findings extend the work of Santana et al. (2022) by specifically identifying mental toughness as a mediator rather than merely a correlate, and complement the developmental perspective of Moore et al. (2021) by demonstrating these relationships in adult university athletes rather than adolescents. Contemporary research on Chinese martial arts has similarly demonstrated that integrated mind-body training enhances both intrinsic motivation and psychological wellbeing in university students (Xu et al., 2026). Recent editorial syntheses have positioned combat sports as powerful vehicles for improving psychological resilience and wellbeing across the lifespan (Ciaccioni et al., 2025). These comparisons situate our findings within a global research context and highlight the unique contribution of our study to the combat sports literature.

### 4.5 Theoretical and practical implications

The findings of this study offer significant implications for both theoretical and applied fields.

**Theoretical implications**: This study enriches Self-Determination Theory (SDT) within the sport psychology context by empirically demonstrating an underlying psychological mechanism-mental toughness-through which intrinsic motivation influences psychological wellbeing. This finding extends SDT by showing that the satisfaction of basic psychological needs (autonomy, competence, relatedness) supports wellbeing not only directly but also indirectly by strengthening an individual's personal resources, particularly mental toughness. Furthermore, our findings contribute to the Broaden-and-Build Theory (Fredrickson, 2001) by illustrating how positive emotions generated through intrinsically motivated activities in combat sports broaden athletes' cognitive and behavioral repertoires, facilitating the accumulation of psychological resources such as mental toughness. The study also advances the growing literature on mental toughness by identifying intrinsic motivation as a key antecedent, thereby integrating motivational and resilience-based frameworks within a unified model. Additionally, the cross-cultural comparisons included in our discussion suggest that the IM-MT-PWB relationship may be influenced by cultural factors, opening avenues for future research examining how collectivist vs. individualist cultural contexts shape these psychological processes.

**Practical implications:** From a practical perspective, the findings support several evidence-based recommendations for coaches, sport psychologists, and sports administrators working with combat sport athletes.

First, coaches should deliberately create an autonomy-supportive environment that nurtures athletes' sources of intrinsic motivation, including curiosity, eagerness to learn, and desire for personal development. This can be achieved by allowing athletes to make autonomous decisions regarding training strategies, providing constructive feedback that reinforces a sense of mastery, and strengthening the sense of belonging within the team through cohesive group activities and positive interpersonal relationships.

Second, alongside physical and technical training, structured programs aimed at developing mental toughness skills should be systematically integrated into training regimens. Evidence-based modules designed to enhance mental toughness-such as goal setting, stress management techniques, cognitive restructuring, and controlled challenge exercises-should be incorporated into regular training cycles. These interventions can help athletes develop the psychological capacities necessary to cope with the unique stressors of combat sports, including performance anxiety, fear of injury, and intense competitive pressure.

Third, given the mediating role of mental toughness identified in this study, interventions targeting both intrinsic motivation and mental toughness may yield synergistic effects. For instance, psycho-educational programs that combine autonomy-supportive coaching practices with mental skills training could be particularly effective in promoting athletes' psychological wellbeing while simultaneously enhancing performance outcomes.

Fourth, sport psychologists and mental performance consultants should consider assessing both intrinsic motivation and mental toughness levels during athlete evaluations to identify those who may benefit from targeted psychological interventions. Early identification of athletes with lower levels of intrinsic motivation or mental toughness could facilitate timely support and prevent potential burnout or psychological distress.

Fifth, at the organizational level, sports administrators and coach educators should incorporate training on motivation and mental toughness development into coaching certification programs. Providing coaches with theoretical knowledge and practical tools to foster intrinsic motivation and mental toughness can create a cascading effect, benefiting athletes across all levels of competition. Additionally, developing institutional policies that prioritize athlete wellbeing alongside performance outcomes can contribute to more sustainable and healthy athletic development.

Sixth, mental toughness training programs can be systematically integrated into regular training cycles through periodized psychological skills training. This includes pre-competition routines (e.g., visualization, arousal regulation), in-season resilience-building exercises (e.g., exposure to controlled stressors, reflective practices), and off-season mental recovery protocols (e.g., mindfulness, psychological detachment). Coaches and sport psychologists should collaborate to design developmentally appropriate interventions that progress from basic coping skills for novice athletes to advanced performance psychology techniques for elite competitors. Such systematic integration ensures that mental toughness development is not treated as an adjunct to physical training but as a core component of holistic athlete development.

These practical recommendations, grounded in empirical findings, offer actionable strategies for enhancing both the psychological wellbeing and performance of combat sport athletes, while also contributing to the broader goal of promoting holistic athlete development.

### 4.6 Limitations and suggestions for future research

When evaluating the findings of this study, certain methodological limitations must be taken into consideration.

First, the sample consists exclusively of university combat sport athletes from Türkiye, which limits the generalizability of the results to other populations, such as elite professional athletes, adolescent practitioners, or athletes from different cultural contexts. While our findings align with research in other individual sports (Gucciardi et al., 2015) and we have discussed potential cultural nuances (Markus and Kitayama, 1991), caution is warranted when extending these results to team sport athletes or non-university populations. The specific demands of combat sports-including high physical contact, individual competition format, and unique psychological stressors-may differ from those in team sports or non-contact individual sports, potentially influencing the relationships among IM, MT, and PWB.

Second, the study's cross-sectional design limits causal inferences and carries the risk of common method variance and social desirability bias. Therefore, longitudinal or experimental studies are needed to confirm the directional relationships suggested by our model.

Third, important variables that could influence the relationships-such as sports experience duration, age, gender, coach-athlete relationship quality, perceived social support, and personality traits-were not controlled.

Fourth, although the Cronbach's alpha for the IM subscale (α = 0.704) exceeded the commonly accepted threshold of 0.70, this relatively modest reliability should be considered when interpreting findings related to intrinsic motivation. However, recent meta-analytic evidence suggests that the 0.70 cutoff is a conventional rather than absolute standard, and that alpha values can vary legitimately across contexts (Hussey et al., 2025). The composite reliability (CR = 0.78) exceeded the recommended threshold of 0.70 (Hair et al., 2019) and provides a more robust estimate of internal consistency. Together with the scale's extensive validation in previous research (Mallett et al., 2007; Demir, 2022), this supports the adequacy of the IM subscale for our analyses.

These limitations provide direction for future research. To test causal relationships, there is a need for experimental or longitudinal designs that measure the effects of psycho-educational interventions focused on IM or MT. For instance, monitoring the effect of psycho-educational intervention programs (intervention group vs. control group) aimed at increasing IM or MT on athletes' PWB over time would provide valuable evidence clarifying the causal direction among variables. To expand the scope of the model, it is recommended to incorporate the Basic Psychological Needs Satisfaction scale into the model to better explain the source of the relationships and to investigate the moderating role of contextual factors such as coach behaviors and team climate. To reduce self-report bias, future studies should adopt multi-method data collection strategies that include both coach evaluations and objective physiological measurements such as heart rate variability (HRV). Finally, to test the cultural and cross-disciplinary validity of the findings, it is recommended that the model be tested with samples from different countries and sport disciplines (e.g., team sports) to establish the boundary conditions and generalizability of our findings.

Several limitations should be considered when interpreting the findings of this study. First, the sample consists exclusively of university combat sport athletes from Türkiye, which may limit the generalizability of the results to other populations, such as elite professional athletes, adolescent practitioners, or athletes from different cultural contexts. While our findings align with research in other individual sports (Gucciardi et al., 2015) and we have discussed potential cultural nuances (Markus and Kitayama, 1991), caution is warranted when extending these results to team sport athletes or non-university populations. The specific demands of combat sports-including high physical contact, individual competition format, and unique psychological stressors-may differ from those in team sports or non-contact individual sports, potentially influencing the relationships among IM, MT, and PWB. Future research should test the proposed model across diverse sport types, competitive levels, and cultural contexts to establish the boundary conditions and generalizability of our findings. Additionally, the cross-sectional design precludes causal inferences, and longitudinal or experimental studies are needed to confirm the directional relationships suggested by our model.

## 5 Conclusion

This study determined that high IM is associated with enhanced PWB in combat sport athletes. Furthermore, the findings indicate that IM strengthens MT and that this enhanced MT plays a significant mediating role in increasing PWB. The study suggests that training environments designed to nurture IM, combined with structured programs that develop MT skills, have the potential to improve not only athletic performance but also athletes' overall psychological health. The findings offer important implications for sustainable athletic success, sport psychology practices, and athlete-centered coaching. Specifically, the results indicate that coaches can enhance both IM and PWB by creating a need-supportive environment (fostering autonomy, competence, and relatedness) and systematically integrating MT training into the training process.

## Data Availability

The raw data supporting the conclusions of this article will be made available by the authors, without undue reservation.
